# Cone beam CT for perioperative imaging in hearing preservation Cochlear implantation – a human cadaveric study

**DOI:** 10.1186/s40463-019-0388-x

**Published:** 2019-11-21

**Authors:** Kayvan Nateghifard, David Low, Lola Awofala, Dilakshan Srikanthan, Jafri Kuthubutheen, Michael Daly, Harley Chan, Jonathan Irish, Joseph Chen, Vincent Lin, Trung Ngoc Le

**Affiliations:** 1 Sunnybrook Research Institute, Sunnybrook Health Sciences Centre, University of Toronto, 2075 Bayview Ave., Room M1, Toronto, 102 Canada; 2 University of Western Australia, School of Surgery, Perth, Australia; 3 Guided Therapeutics (GTx) Program, TECHNA Research Institute, University of Toronto, University Health Network, Toronto, Canada; 4 Department of Otolaryngology-Head and Neck Surgery/Surgical Oncology, Princess Margaret Cancer Centre/University Health Network, Toronto, Canada

## Abstract

**Background:**

Knowledge of the cochlear implant array’s precise position is important because of the correlation between electrode position and speech understanding. Several groups have provided recent image processing evidence to determine scalar translocation, angular insertion depth, and cochlear duct length (CDL); all of which are being used for patient-specific programming. Cone beam computed tomography (CBCT) is increasingly used in otology due to its superior resolution and low radiation dose. Our objectives are as followed:

1. Validate CBCT by measuring cochlear metrics, including basal turn diameter (A-value) and lateral wall cochlear duct length at different angular intervals and comparing it against microcomputed CT (uCT).
2. Explore the relationship between measured lateral wall cochlear duct length at different angular intervals and insertion depth among 3 different length electrodes using CBCT.

**Methods:**

The study was performed using fixed human cadaveric temporal bones in a tertiary academic centre. Ten temporal bones were subjected to the standard facial recess approach for cochlear implantation and imaged by CBCT followed by uCT. Measurements were performed on a three-dimensional reconstructed model of the cochlea. Sequential insertion of 3 electrodes (Med-El Flex24, 28 and Soft) was then performed in 5 bones and reimaged by CBCT. Statistical analysis was performed using Pearson’s correlation.

**Results:**

There was good agreement between CBCT and uCT for cochlear metrics, validating the precision of CBCT against the current gold standard uCT in imaging. The A-value recorded by both modalities showed a high degree of linear correlation and did not differ by more than 0.23 mm in absolute values. For the measurement of lateral wall CDL at various points along the cochlea, there was a good correlation between both modalities at 360 deg and 720 deg (r = 0.85, *p* < 0.01 and r = 0.79, p < 0.01). The Flex24 electrode displayed consistent insertion depth across different bones.

**Conclusions:**

CBCT reliably performs cochlear metrics and measures electrode insertion depth. The low radiation dose, fast acquisition time, diminished metallic artifacts and portability of CBCT make it a valid option for imaging in cochlear implant surgery.

## Introduction

Cochlear implantation (CI) is a significant breakthrough in the auditory rehabilitation of patients with bilateral severe to profound hearing loss [1, 2]. More recently, there has been an expansion of implant candidacy to include patients with residual hearing. In these patients, the use of electroacoustic stimulation mandates that low frequency regions of the cochlea are preserved anatomically and functionally to facilitate acoustic stimulation, while the high frequency regions are stimulated electrically [3].

In addition to atraumatic surgical technique, surgeons have resorted to shorter, or slimmer and softer electrodes to preserve the low frequency regions of the cochlea. Although over-insertion of the electrodes leads to injury, under-insertion may result in incomplete cochlear coverage, inadequate stimulation and compromised functional outcomes [4, 5]. As there is a known variation in cochlear duct length (CDL), knowledge of the anatomy of the cochlear duct and position of the CI electrode in these patients will allow implant surgeons to customize electrode choice and insertion depth to improve functional outcomes [2, 6].

Postmortem histological examination is the gold standard for assessing the properties and positions of CI electrodes in cadaveric cochleae [7]. However, this technique is not feasible clinically. As such, there has been an emphasis of the development of in vivo imaging techniques and image processing. The standard high resolution multi-slice CT scan of the temporal bone is limited by spatial resolution and metallic artifacts. Cone beam computed tomography (CBCT) is a relatively new in vivo imaging procedure that was first developed for dental and maxillofacial examinations in 1998 [8]. This imaging technique allows for fast acquisition, visualization of high contrast structures with superior resolution, reduction of metallic artifacts, and has a lower dose of radiation at the expense of soft tissue enhancement and resolution [2, 9]. These characteristics make CBCT an ideal imaging modality for preoperative planning and post-insertion imaging in cochlear implantation. Its portability also makes it feasible to use in the intraoperative setting.

Microcomputed CT (uCT) produces images with unsurpassed spatial resolution and reduced metallic artifact in cadaveric specimens and live animal research. However, application of uCT in the clinical setting is not practical due to large size of gantry, small sample capacity, high levels of radiation, high cost, limited availability and poor portability [9].

Our objectives are as followed:

1. Validate CBCT by measuring cochlear metrics, including basal turn diameter (A-value) and lateral wall cochlear duct length at different angular intervals and comparing it against microcomputed CT (uCT).
2. Explore the relationship between measured lateral wall cochlear duct length at different angular intervals and insertion depth among 3 different length electrodes using CBCT.

## Materials and methods

### Preparation of temporal bones

Ten fixed human cadaveric temporal bones were acquired from the Division of Anatomy, University of Toronto, in compliance with the Anatomy Act of Ontario and Institutional Research Ethics Board protocols (#09–0751). A standard approach for CI, consisting of a cortical mastoidectomy, posterior tympanotomy (facial recess approach) and round window exposure was performed on each bone.

### Acquisition of Images

CBCT was performed using a prototype mobile C-arm for intraoperative three-dimensional (3D) CBCT imaging. This prototype was developed in collaboration with Siemens Healthcare (Erlangen, Germany) with key modifications including the addition of a flat panel detector, motorized orbit and software control system. Previous image-guided surgery studies with this system include image quality assessments in pre-clinical temporal bones, [7, 10] and a clinical head & neck surgery trial [11]. CBCT scans were reconstructed to encompass a 20 × 20 × 15 cm field of view with isotropic voxel dimensions of 0.2 mm [12]. X-ray tube peak voltage and current was set at 100kVp and 2.6 mA.

Next, uCT was performed on a GE Locus Ultra (GE Healthcare, Waukesha, WI), a dedicated pre-clinical small animal scanner. Volumetric uCT images for this study encompassed 12 × 12 × 10 cm with isotropic 0.15 mm 3D voxels, and 80kVp and 50 mA for image acquisition.

### Image processing and measurements

Multiplanar reconstruction was then performed and the images were viewed on the in-house 3D visualization software (GTx-Eyes), which is based on open-source toolkits (e.g. IGSTK [13], VTK [14], ITK [15]). A semi-automated algorithm within the software performed segmentation of the cochlea from the surrounding otic capsule and the segmented data was used to create a 3D model of the cochlea.

**Fig. 1:**
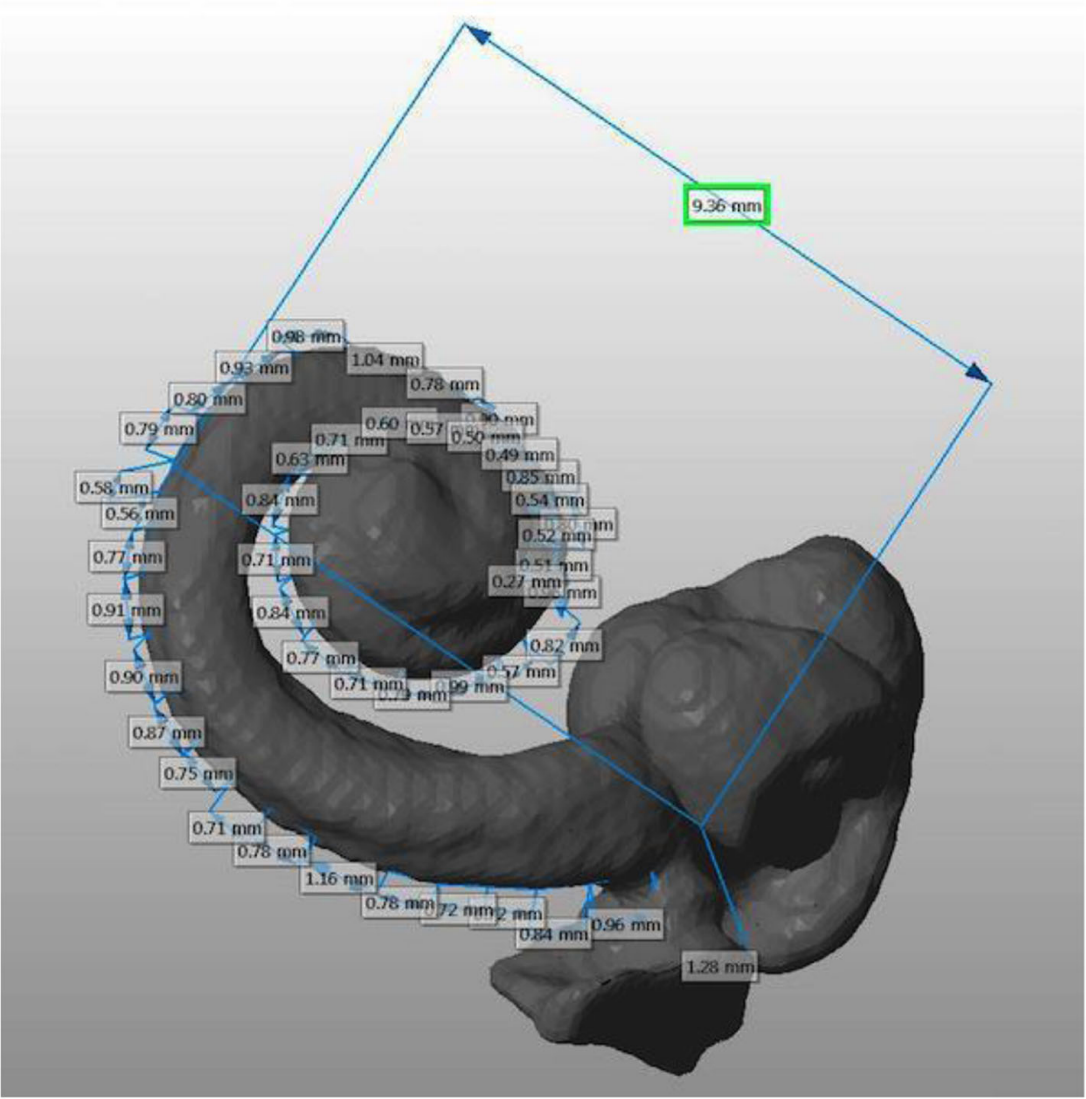
3D mesh model of cochlea segmented from CBCT scan used to measure cochlear duct length and basal diameter. Incremental measurements displayed in millimeter from the round window, along the lateral wall of cochlear duct length, for the first two turns of the cochlea (360 and 720 degrees)

Netfabb Studio Basic 4.9, a standard 3D modeling software, was then used to analyze this reconstructed model. Measurements were performed manually using the provided measurement tools (see Fig. 1). For each bone, the following parameters were measured: the diameter of the basal turn of the cochlea (A-value) and the sequential length of the cochlear lateral wall from the round window to the completion of two cochlear turns (360^o^, 450^o^, 540^o^ and 720^o^), with the lateral wall location defined as the furthest point from centre at any given point along the cochlea [16]. The apex was not reliably well-visualized on the scans and hence was not included in subsequent measurements. For the measurement of the diameter of the basal turn of the cochlea, the point along the lateral wall of the basal turn that had the furthest straight-line distance from the round window was chosen. The A-value was then defined as the distance between this point and the mid-point of the round window. A line drawn through the round window and the centre of the modiolus defined the start and end point of each turn of the cochlea [17].

### Study of electrode insertion depth

The sequential placement of 3 Med-El (Med-El GmbH, Innsbruck, Austria) electrodes of different lengths was then performed in 5 human cavaderic bones. The three electrodes used were the Flex24 (24 mm), Flex28 (28 mm), and FlexSoft (31.5 mm), which had active stimulation lengths of 20.9 mm, 23.1 mm, and 26.4 mm, respectively. These electrodes were inserted through the posterior tympanotomy and into the scala tympani via an incision on the round window membrane. Insertion was stopped when resistance was encountered to avoid trauma and to emulate structural preservation techniques. Measurements were made for each of the 3 electrodes in each of the 10 temporal bones, with the shortest electrode placed first. Electrode insertion depth was measured along the lateral wall from the round window to the tip of the electrode.

A CBCT of these temporal bones with the electrodes in place were then performed. The images were processed and analyzed in a similar fashion as described above.

### Statistical analysis

IBM® SPSS® software (version 23.0. Armonk, NY: IBM Corp) was used to analyze the acquired data. Correlation between both modalities for A value and lateral wall CDL was quantified by calculating the absolute difference and utilizing Pearson’s correlation. A *p* value of less than 0.05 was considered to be statistically significant.

## Results

**Fig. 2:**
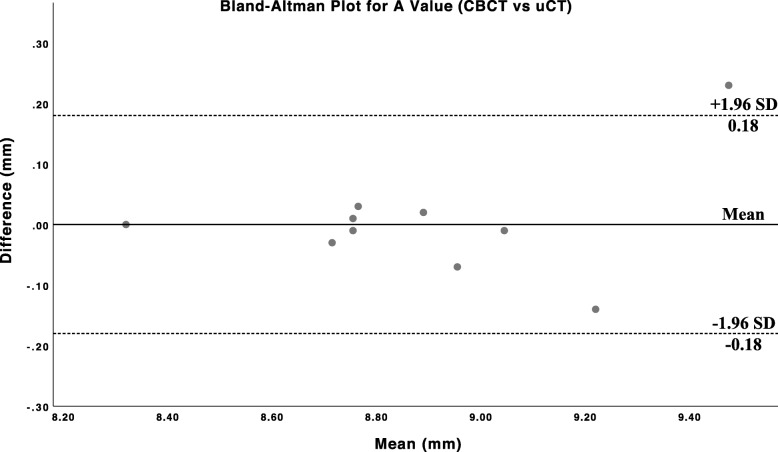
Bland-Altman plot of calculated differences in A-values obtained with CBCT and uCT. Solid line represent bias or mean. Dotted lines at ±0.18 mm represent upper and lower limits of agreement at 1.96 standard deviations

Ten cadaveric human temporal bones were used for our study, with 6 right-sided bones and 4 left-sided bones. The differences in A-values as measured by CBCT and uCT were compared in Fig. 2. The means and standard deviations for A-value using CBCT and uCT were 8.89 ± 0.33 mm, and 8.89 ± 0.30 mm, respectively. When both modalities were compared, the largest absolute difference in A-values obtained was 0.23 mm. There was a good linear correlation between A-values measured through both modalities (r = 0.96, *p* < 0.01).

**Fig. 3:**
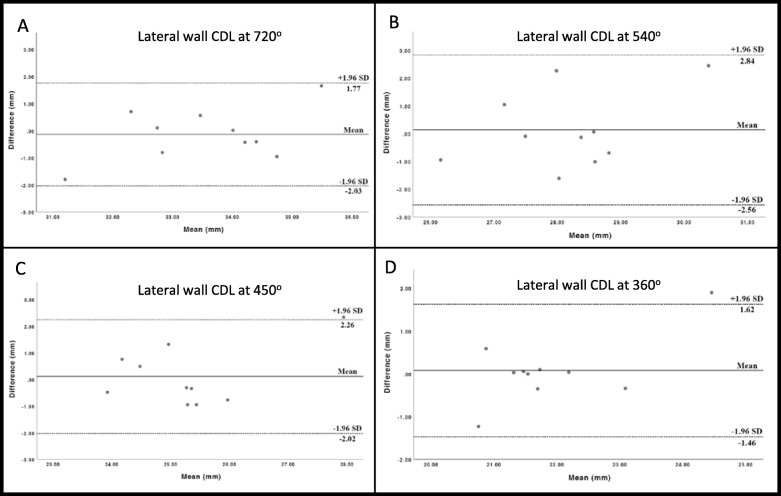
Bland-Altman plots of calculated differences in lateral wall cochlear duct lengths at various angular intervals obtained with CBCT and uCT. Panels A-D depict CDL at 720^o^, 540^o^, 450^o^, 360^o^ respectively. Solid line represent bias or mean. Dotted lines represent upper and lower limits of agreement in millimeter at 1.96 standard deviations

Differences in lateral wall CDLs for both modalities at various angular intervals along the cochlea are compared in Fig. 3. The means and standard deviations of these measurements are summarized in Table 1. Table 1 also provided a summary of differences in A-value, and CDL at various intervals of 360^o^, 450^o^, 540^o^, 720^o^ comparing between modalities (CBCT vs uCT). The mean, standard deviation, mean absolute difference, absolute difference range showed no difference between two modalities with *p* values well above 0.05. When lateral wall CDL for both modalities were compared in Table 2, measurements at 360^o^ and 720^o^ showed the most significant linear correlation (r = 0.85, *p* < 0.01 and r = 0.79, *p* < 0.01 respectively).

**Table 1:** Summary of descriptive statistics for A-value, and lateral wall cochlear duct lengths at various angular intervals along the cochlea.

Modality	Descriptive Test	A-value (mm)	720° (mm)	540° (mm)	450° (mm)	360° (mm)
uCT	Mean	8.89	33.61	28.09	25.22	21.87
	Standard Deviation	0.30	1.14	1.07	1.03	0.90
CBCT	Mean	8.89	33.48	28.28	25.34	21.95
	Standard Deviation	0.33	1.57	1.49	1.44	1.41
CBCT vs uCT	Mean Absolute Difference	0.06	0.74	1.03	0.87	0.46
	Absolute Difference Range	0.00–0.23	0.02–1.79	0.07–2.45	0.30–2.35	0.00–1.90
	*p*-value	0.92	0.68	0.76	0.73	0.76

Measurements were obtained and compared between CBCT vs uCT modalities. Significant *p*-values are defined as < 0.05

**Table 2:** – Correlation of cochlear duct length derived from uCT and CBCT at various angular intervals

Degrees	Pearson Correlation coefficient	p value
720	0.79	< 0.01
540	0.48	0.161
450	0.65	0.020
360	0.85	< 0.01

Table 3 tabulates the lateral wall CDL and electrode insertion depths for each of the 3 different electrodes utilized in the other 5 human temporal bones. An estimated organ of Corti length was calculated to be about 0.87185 times of the lateral wall CDL [18]. This estimated organ of Corti length was provided as a more relevant metric or “correction factor” when comparing with the electrode insertion depths. Although the insertion depth for the Flex24 electrode was consistent across different temporal bones, there was no observable relationship between the depths of the other 2 electrodes.

**Table 3:** – Comparison between insertion depths of Flex 24, Flex 28, and FlexSoft electrodes and lateral wall cochlear duct lengths at various angular interval for bones A1–5

Bone	Lateral wall CDL measured (mm)		Organ of Corti length estimated (mm)		Electrode Insertion depth measured (mm)		
	360°	720°	360°	720°	Flex24	Flex28	FlexSoft
A1	21.4	34.1	18.7	29.7	24.0	26.0	26.1
A2	21.8	34.2	19.0	29.8	24.0	28.8	27.0
A3	22.4	34.0	19.5	29.6	24.0	27.1	27.1
A4	21.5	33.0	18.7	28.8	24.0	26.1	26.3
A5	23.2	34.4	20.2	30.0	24.0	31.0	31.0

Estimated organ of Corti lengths at various angular intervals were calculated to be (0.87185 x lateral wall CDL), an estimation from Kawano et al. [18]

## Discussion

Our study has shown that there is good agreement between CBCT and uCT for cochlear metrics, noting that the latter is considered to be the current gold standard in imaging [6]. The A-value recorded by both modalities showed a high degree of linear correlation and did not differ by more than 0.23 mm in absolute values, as demonstrated in Table 1 and Fig. 1. For the measurement of lateral wall CDL at various points along the cochlea, there was a good correlation between both modalities at 360^o^ and 720^o^. Unexpectedly, the correlation at 450^o^ and 540^o^ was poorer. This was likely due to the technique through which the measurements were performed on the segmented 3D models. As the various points along the cochlea were defined relative to a line drawn through the centre of the round window and modiolus, a greater variability can be expected for points that were further away from both these landmarks. We postulate that automation of the process will lessen this variability in the future.

Notwithstanding the necessary refinements, we expect CBCT to be of use for preoperative planning, especially when performing hearing preservation CI surgery. In this form of surgery, the importance of preserving residual hearing in the lower frequencies mandates that the electrode must not be over-inserted. Under-insertion, on the other hand, may lead to inadequate stimulation and poorer functional outcomes. When considered with the preoperative audiogram, coupled with manual or automated measurements of typical A-value and CDL [19–23], preoperative imaging allows the surgeon to select the electrode with the most appropriate length for the patient’s CDL and configuration of hearing loss [7, 24–26]. For this purpose, the CBCT is advantageous to conventional CT because of its accuracy [27], lower dose of radiation [28] and faster acquisition time [9]. CBCT can acquire all projections in the single rotation, and thus the acquisition of results is relatively rapid [9]. In comparison to conventional CT, the CBCT provides significant dose reductions, more specifically between 98.5 and 76.2% [9, 28–30].

Our study also demonstrates that it is feasible to use CBCT to measure electrode insertion depths for post-operative frequency mapping. This is supported by other studies, some of which even suggest that it is superior to conventional CT because of its lower propensity for metallic artifact [9]. This property coupled with its portability and fast acquisition time, makes CBCT an ideal postoperative imaging modality for confirmation of electrode position and measurement of insertion depth for individualized programming and frequency mapping. The case for accurate postoperative knowledge of electrode insertion depth is made by our results in Table 3 when measured using CBCT. All three electrodes consistently reach 360^o^ angular interval in all 5 bones. However, the longer electrodes only reach 720^o^ in 1 out of 5 bones, confirming there may be situations in smaller cochleae where a shorter electrode should be used. Furthermore, shorter electrode (Flex24) has more predictable insertion depth across the bones while longer electrode (Flex28 and FlexSoft) insertion depths do not obey a consistent relationship with lateral wall CDL, organ of Corti length, or electrode lengths. This could be a result of inconsistent positions of different segments of the electrode in the second turn (vary from lateral wall to mid-scala to modiolus), and the soft nature of Med-El electrode [31]. Thus, fine adjustments will likely have to be made to postoperative frequency mapping based on the electrode position information obtained from postoperative imaging. Hence, CBCT could also be used for postoperative imaging for patient-specific custom frequency maps due to its high-resolution electrode localization and low metal artifact [32].

The first strength of our study is its originality in providing detailed cochlear metrics using a simple and quick CBCT scan. The resulting metrics have shown to be highly correlated with those obtained from the high-resolution μCT and hence can be a validated framework for future automation. Another strength of our study is its clinical relevance, using different electrode lengths to demonstrate poor prediction value of lateral wall CDL and insertion depth at various angular intervals of the cochlea. Our goal was not to compare CBCT with conventional CT given the current limitations of conventional CT in regards to metallic artifacts and high dose of radiation. We are set to validate the value of CBCT in comparison to that of uCT which is considered to be the gold standard in terms of spatial resolution [6]. Consistently, other studies comparing CBCT to conventional CT [33], and histology [34] have been done to indirectly support our findings that CBCT is a highly reliable modality for cochlear metrics.

The main limitations of our study are the small sample size, potential single interpreter error, and the use of cadaveric temporal bones, which may not be reflective of in vivo imaging. For the second part of our study, sequential insertion of the 3 different electrodes in the same temporal bone may have altered intracochlear anatomy and contributed to inconsistencies in insertion depth for the latter 2 electrodes. An additional limitation was that uCT imaging was performed on a 154 μm scanner, as opposed to a higher-resolution scanner (e.g. 20–50 μm) that may have provided a better gold-standard for comparison. Specialized 3D segmentation, semi-automation, and modeling softwares (such as GTx-Eyes or Netfabb Studio or similar) might not be widely available. Manual labour and time were also required for the extraction of imaging data, preprocessing and performing measurements. However, with further development, it is likely that these processes can be automated and incorporated into the standard radiological viewing software [35].

## Conclusion

This study has shown that CBCT has sufficient spatial resolution to accurately and reliably determine cochlear metrics including A-value, lateral wall cochlear duct length and electrode insertion depth. With low radiation dose, fast acquisition time, low propensity for metallic artifact and portability, CBCT should be considered in lieu of conventional CT for cochlear implant imaging.

## Data Availability

Available.

## Abbreviations

3D: three dimensions
CBCT: cone beam computed tomography
CDL: cochlear duct length
CI: cochlear implant
Flex 24, 28, Soft (31.5): different electrode lengths from Med-El GmbH
μCT: microcomputed tomography
